# Workplace experiences of mental health consumer peer workers in New South Wales, Australia: a survey study exploring job satisfaction, burnout and turnover intention

**DOI:** 10.1186/s12888-020-02688-9

**Published:** 2020-06-01

**Authors:** Justin Newton Scanlan, Megan Still, Jae Radican, Daya Henkel, Tim Heffernan, Peter Farrugia, Jemima Isbester, Jessica English

**Affiliations:** 1 Present Affiliation: Faculty of Medicine and Health, The University of Sydney, Sydney School of Health Sciences, Office J120, Cumberland Campus, 75 East Street, Lidcombe, Sydney, NSW 2141 Australia; 2 Present Affiliation: Mental Health Services, Sydney Local Health District, Concord, NSW Australia; 3 Present Affiliation: Official Visitors Program, Gladesville, NSW Australia; 4 Previous affiliation (at the time of the study): NSW Ministry of Health, Mental Health Branch, North Sydney, NSW Australia; 5 Present Affiliation: NSW Ministry of Health, Mental Health Branch, Chatswood, NSW Australia; 6 Previous affiliation (at the time of the study): Hunter New England Local Health District, Mental Health Services, Newcastle, NSW Australia; 7 Present Affiliation: COORDINARE – South Eastern NSW Primary Health Network, Wollongong, NSW Australia; 8 Present Affiliation: Flourish Australia, Sydney Olympic Park, NSW Australia; 9 Present Affiliation: ReachOut Australia, Sydney, Australia; 10 Previous affiliation (at the time of the study): Being: Mental Health and Wellbeing Consumer Advisory Group, Sydney, NSW Australia

**Keywords:** Peer worker, Consumer worker, Consumer peer worker, Consumer, Lived experience practitioner, Burnout, Stigma, Workforce, Mental health services

## Abstract

**Background:**

Consumer peer workers are individuals with lived experience of mental health issues and recovery who are employed to use their lived experience to support others. The consumer peer workforce has expanded substantially in recent years. While some research has explored the workplace experiences of peer workers, no previous studies have explored job satisfaction, burnout or turnover intention for this workforce.

**Methods:**

Consumer peer workers in New South Wales, Australia were invited to complete a survey designed to explore their workplace experiences. The survey included measures of job satisfaction, burnout, turnover intention, job demands and job resources, and satisfaction with supervision, professional development and opportunities for career progression. Questions also explored positive and negative aspects of positions. Analyses included exploration of the relationships between of job satisfaction, burnout, turnover intention, job demands and job resources as well as tabulation of common positive and negative aspects of positions. Results were also compared with findings from a previous study exploring workplace experiences of other mental health workers.

**Results:**

A total of 67 peer workers participated in the study. Overall job satisfaction, burnout (disengagement and exhaustion) and turnover intention for peer workers was not significantly different to other mental health workers. Job satisfaction, disengagement, exhaustion and turnover intention were all significantly inter-related. Job resources of social support, job control, feedback, and rewards and recognition were associated with positive workplace experiences and the job demand of “physical environment” was most substantially associated with poorer workplace experiences. The most common positive aspect of positions was “connecting with consumers” and the most common negative aspect of positions was “attitudes of clinicians / workplace culture”. Access to supervision from a senior peer worker was associated with more positive workplace experiences.

**Conclusions:**

This research demonstrates that while consumer peer workers do not appear to experience poorer job satisfaction or higher levels of burnout or turnover intention than other mental health workers, a range of challenges do exist. Efforts to further expand the peer workforce (especially senior peer worker roles) and to promote more positive attitudes and workplace cultures are likely to promote better workplace experiences for peer workers.

## Background

Peer workers in mental health services are people with lived experience of mental health issues and recovery who are employed to use their lived experience to support consumers who access mental health services [1]. While the value of consumer involvement in the design, delivery and evaluation of mental health services has been recognised in Australian policy for several decades [2], there has been significant growth in the peer workforce in Australia in recent times [1]. International evidence demonstrates the benefits of peer work, including improvements in engaging consumers in caring relationships, improving relationships between consumers and outpatient providers, increasing engagement in non-acute and less costly care, decreasing substance use, decreasing unmet needs, increasing hope, empowerment, self-efficacy, social functioning, quality of and satisfaction with life, and engagement with self-management strategies [3–9].

Despite the benefits that are provided by peer workers within the mental health workforce, peer workers have identified a range of challenges associated with their role. One of the most commonly-reported set of challenges relates to attitudes of non-peer mental health staff. Some staff may struggle to form collegiate relationships with peer workers, rather perceiving peer workers as “patients”, interns or pseudo-staff and others don’t value the contribution of peer support in a mental health context [10–13]. Stigma, discrimination and isolation have also been identified, often related to perceptions that peer workers are “emotionally fragile” and may be unable to cope with the emotional stressors of work in mental health services [4, 14–16]. Workplace cultures that are not aligned with recovery and non-strengths-based language used by staff when discussing consumers can create stress, dissatisfaction and a sense of disconnection between peer and non-peer staff [10, 13, 16–18].

Other issues relate to the design of services. As peer workers make up a relatively small proportion of the workforce, they often work solo in teams without other peer workers. This can lead to a lack of support from, and connection with, other workers which can create a sense of isolation, inferiority and invisibility, particularly where the role is not valued by other members of the team [12, 13, 15, 19]. Finding appropriate supervision and mentoring from experienced peer workers due to the lack of senior peer worker roles and peer supervisor roles can also be challenging [10, 12, 15, 17, 20]. The role of peer workers is often poorly articulated within services and this can cause role confusion and conflict and can result in peer workers taking on menial tasks that other workers do not wish to do [4, 12, 15, 16, 18, 19, 21].

High workloads, job insecurity, lack of hours, disparity in pay and lack of opportunities for career advancement have also been identified as issues [13, 15–17, 22]. Other challenges relate to peer workers managing their own mental wellbeing in relation to maintaining appropriate standards of work and observing, enforcing and respecting boundaries when developing relationships with consumers, as well as supporting consumers in distress [15–18].

Finally, peer workers often view their roles as “trail blazing” and being responsible for changing the culture of mental health services [10, 16, 20, 23]. Without sufficient leadership roles for peer workers, this role can be challenging and may not effect change [7, 10, 20, 24, 25].

In Australia, peer workers are employed in a range of different mental health services across different service sectors. However, the majority are employed in either specialist public mental health services operated by state government departments of health or community managed organisations funded to provide psychosocial support services to individuals living with mental illness [1]. Government and community managed services tend to be structured differently, with government mental health services employing mainly professionally-qualified staff (nurses, occupational therapists, psychiatrists, psychologists and social workers) and community managed organisations employing a more diverse range of individuals with a variety of professional and para-professional qualifications and experience [26]. Given these different structures, community managed organisations have tended to employ a larger proportion of individuals with lived experience of mental health issues and, therefore, may be better positioned to engage and support the expanding peer workforce [11, 17, 27].

Despite expanding numbers of peer workers and the range of challenges that have been identified, there has been limited research into the work-related experiences of peer workers, especially in terms of commonly-used measures such as job satisfaction, burnout and turnover intention. Given this paucity of research, this study was established to explore workplace experiences and job satisfaction, turnover intention and burnout in peer workers working in mental health services in New South Wales, Australia. The specific research questions guiding this study were:

1. What are the factors that attract individuals to apply for peer worker positions?
2. What are the aspects of work that peer workers like and dislike?
3. What aspects of work lead peer workers to consider leaving their positions?
4. How satisfied are peer workers with supervision, professional development and opportunities for career progression?
5. What levels of job satisfaction, turnover intention and burnout are reported by peer workers and how does this compare with other groups in the mental health workforce?
6. What are the relationships between various aspects of work experienced by peer workers and job satisfaction, turnover intention and burnout (disengagement and exhaustion)?
7. Are there differences in experiences for those peer workers employed in government and community managed services?

## Method

The research team included six researchers with lived experience of mental health issues and recovery who were working in various peer-work related roles (peer worker, senior peer worker, peer work coordinator and state-wide peer work coordinator). The remaining two researchers did not identify as having lived experience of mental health issues and recovery, but had been involved in supporting the expansion of peer work initiatives in mental health services as well as in previous consumer-led and collaborative research projects. These two researchers also had previous experience in research projects exploring the work-related experiences of other professional groups in mental health. As a collaborative research project, special efforts were made to ensure that peer worker researchers’ perspectives were given primacy in decision-making processes [28]. This study was approved by The University of Sydney Human Research Ethics Committee (reference number: 2017/886).

### Survey distribution and management

#### Survey hosting

Study data were collected and managed using REDCap electronic data capture tools hosted at The University of Sydney [29].

#### Sampling frame

Inclusion criteria for the study were: (a) currently working in an identified consumer peer worker role and (b) currently working in the state of New South Wales, Australia. Individuals with a lived experience of mental illness working in other positions within mental health services were excluded as it was expected that the experiences of individuals working in non-peer defined roles would likely be different from the experiences of individuals in peer-defined roles. Determining the complete sample frame was challenging as the specific number of consumer peer workers in New South Wales was not available. Estimates for peer workers working in government mental health services in New South Wales in 2018 ranged from 34.5 full time equivalent (FTE) [30] to 100 FTE [31]. No data were available relating to the number of identified consumer peer worker positions in the community-managed mental health sector.

#### Survey distribution and data collection

Information about the study was distributed via email through an established network of peer workers and the state-wide consumer advocacy organisation. Data were collected between January and April 2018. Participants did not receive any monetary or other compensation for completing the survey.

### Instrumentation

To allow comparison between peer workers and other members of the mental health workforce, the questionnaire was based on ones used in previous mental health workforce surveys [32–34]. The questionnaire included a range of demographic and work-related questions as well as a suite of open-ended questions and scales designed to explore peer workers’ work-related experiences. These questions and scales are described below.

#### Reasons for applying for current position, enjoyable aspects of role and disliked aspects of the role

Participants were asked three open ended questions related to these topics. These were: (a) What were the reasons you decided to apply for your current position?; (b) Please list any factors that you like about your current position that keep you coming back to work each day; and (c) Please list any factors that you do not like about your current position. In the context of this study, it was determined that having open-ended questions was more beneficial than using a pre-determined list of factors. Taking the open-ended approach meant that participants could list any factors that were personally relevant.

#### Satisfaction

Overall job satisfaction was rated on a single-item 10-point scale. Participants were asked “Overall, how satisfied are you with your current job?” Response anchors were (1) Very dissatisfied; (5) and (6) Neither satisfied nor dissatisfied; and (10) Very satisfied. While there is debate surrounding whether single-item or multiple-item scales of job satisfaction are superior, previous research has demonstrated the validity of single item measures [35–37].

In addition to overall job satisfaction, participants were also asked to rate their level of satisfaction with three specific aspects of their work: supervision, access to professional development and opportunities for career progression. These were rated on a 5-point scale from “Very dissatisfied” (scored 1) to “Very satisfied” (scored 5). These items have been used in a previous study [32], but have not been formally evaluated for validity or reliability. Although not specifically designed to be used as a scale, the internal consistency of these three items (Cronbach’s alpha) was 0.66. In relation to the supervision question, participants also identified whether they received supervision (as distinct from management) from a senior peer worker, another peer worker at the same level, a mental health worker or “other”.

#### Turnover intention

Turnover intention was measured by three statements: “I am actively looking for another job,” “As soon as I find another job, I will quit” and “I often think about quitting my job” [38]. Each had three response options: (1) No; (2) Unsure; and (3) Yes. Overall turnover intention was an average of the three responses, with higher scores indicating higher levels of turnover intention. Internal consistency (Cronbach’s α) was 0.82.

In addition to these questions, if participants responded with “Unsure” or “Yes” to any of the questions, they were invited to give the reasons why they were considering quitting.

#### Burnout

Burnout was measured using the Oldenburg Burnout Inventory (OLBI) [39, 40]. The OLBI measures two dimensions of burnout: disengagement and exhaustion. Disengagement refers to distancing one’s self from one’s work and having negative feelings towards one’s work or service recipients and exhaustion refers to the depletion of physical and emotional energy [41, 42].

Although the Maslach Burnout Inventory (MBI) [43] is a more commonly-used measure of burnout, the OLBI was selected for this study as it is aligned with the Job Demands-Resources model of burnout [41, 42] which guided the development of the survey used in this study. Additionally, as it includes both positively and negatively worded items, it is suggested to have superior psychometric properties when compared to the MBI [39].

Scoring of the OLBI followed the recommended approach [40] with scoring for negatively-worded items reversed. In this context, higher scores represent higher levels of disengagement or exhaustion. In this study, internal consistency (Cronbach’s α) was 0.78 for disengagement and 0.82 for exhaustion.

#### Job demands and job resources

Using the Job Demands-Resources model [41, 42] as its basis, job demands and job resources were evaluated using a questionnaire developed for previous studies [32, 33]. Job demands are work characteristics that can cause stress or require the exertion of cognitive, emotional or physical energy. Job resources are external factors that provide support to employees. The questionnaire included 25 questions related to nine types of job demands (cognitive, emotional, physical workload, physical environment, recipient contact, shiftwork / working hours, time pressure, work-home interference and workload) and eight job resources (feedback, job control, job security, participation, rewards and recognition, social support, supervisor support and manager support). In this study, internal consistency (Cronbach’s α) was 0.79 for job demands and 0.84 for job resources.

### Analyses

Responses to open-ended questions were coded inductively using principles of thematic analysis [44]. Each response was coded according to the ideas presented in the response. As participants frequently presented several ideas in their responses to these questions, up to eight codes were allocated to each response. Initially, two researchers (MS and JNS) independently coded responses to each question and then compared these codes via discussion. The codes developed via this process (especially those where consensus was not reached between MS and JNS) were then checked, expanded and revised by several other members of the research team. This was initially done separately and then together in a coding meeting where individual codes were discussed until consensus was achieved. The final set of codes were discussed and agreed to at a research team meeting.

Quantitative analyses were completed using IBM SPSS Statistics, Version 25. The influence of missing data was minimised in this study through the online survey setup requiring responses for most fixed-choice questions. Analyses completed for each research question are described below:

*1. What are the factors that attract individuals to apply for peer worker positions?; 2. What are the aspects of work that peer workers like and dislike?; and 3. What aspects of work lead peer workers to consider leaving their positions?*

Frequencies of themes identified in the qualitative analysis from responses to the relevant open-ended questions for each of these areas were calculated and tabulated.

*4. How satisfied are peer workers with supervision, professional development and opportunities for career progression?*

Descriptive statistics (means and standard deviations) were calculated for each of these responses. Additionally, a one-way ANOVA was completed to examine whether there were differences in level of satisfaction with supervision according to the type of person providing the supervision.

*5. What levels of job satisfaction, turnover intention and burnout are reported by peer workers and how does this compare with other groups in the mental health workforce?*

Initially, means and standard deviations for job satisfaction, turnover intention and burnout (disengagement and exhaustion) were calculated. These were then compared with results from a previous study [33] using independent-samples *t*-tests. The previous study was completed in a large (government) metropolitan mental health service in New South Wales with 21 inpatient units, 10 community health centres and approximately 1100 clinical staff [33]. A total of 277 clinicians participated in the study, including 43 medical staff, 123 nurses, 34 occupational therapists, 32 psychologists, 26 social workers and 19 “other” / not stated [33].

*6. What are the relationships between various aspects of work experienced by peer workers and job satisfaction, turnover intention and burnout?*

Bivariate correlation coefficients were calculated between job satisfaction, turnover intention, disengagement and exhaustion and the various job demands, job resources and reported satisfaction with supervision, professional development and opportunities for career progression.

*7. Are there differences in experiences for those peer workers employed in government and community managed services?*

As government services and community managed organisations are structured differently, we hypothesised that the experiences of consumer peer workers within these two sectors might be different. Independent *t*-tests were completed to compare ratings of job satisfaction, turnover intention, burnout and satisfaction with supervision, professional development and career progression opportunities between peer workers employed in government and community managed organisations. Additionally, responses from peer workers employed in government and community managed services for questions related to reasons for applying for positions, likes, dislikes and reasons for considering leaving their positions were tabulated and explored for significant differences using Pearson’s chi-square statistic.

## Results

### Participants

A total of 67 peer workers participated in the study. The majority of these participants (*n* = 46, 68.7%) worked in government services, 20 (29.9%) worked in community managed organisations and one (1.5%) worked in the private sector. Respondents from government services worked an average of 28.1 h per week (0.74 full time equivalent [FTE]). This equates to 34.0 FTE responses. Using existing estimates for numbers of peer workers in government services [30, 31], this response rate equates to somewhere in the range of 34 to 98% for government service respondents. Demographic and work-related variables are summarised in Table 1.

**Table 1:** Demographics of the sample (*N* = 67)

Domain	Characteristic	Freq.	Percent
Gender identity	Female / Woman	47	70.1%
	Male / Man	18	26.9%
	Transgender Man	2	3.0%
Sector	Government service (New South Wales Health)	46	68.7%
	Community Managed Organisation	20	29.9%
	Private Sector	1	1.5%
Job Title	Peer Worker / Peer Support Worker	45	67.2%
	Senior Peer Worker / Senior Peer Support Worker	4	6.0%
	Consumer Advocate	3	4.5%
	Health Peer Support Worker / Peer Health Coach	4	6.0%
	Other^a^	10	14.9%
	Not stated	1	1.5%
Main work location	Metropolitan	32	47.8%
	Regional	18	26.9%
	Rural	14	20.9%
	Remote	3	4.5%
Current employment type	Permanent	47	70.1%
	Temporary / Fixed-term contract	18	26.9%
	Casual	2	3.0%
Focus of current position	Totally / almost totally community-based	33	49.3%
	Mainly community but some inpatient work	10	14.9%
	About an even split between community and inpatient	6	9.0%
	Mainly inpatient, but some community work	8	11.9%
	Totally / almost totally inpatient-based	10	14.9%
Length of time working in current position	Less than 1 year	18	26.9%
	1–2 years	22	32.8%
	3–5 years	17	25.4%
	6–10 years	4	6.0%
	11–20 years	4	6.0%
	Over 20 years	2	3.0%
Length of time working in peer work positions	Less than 1 year	12	17.9%
	1–2 years	15	22.4%
	3–5 years	21	31.3%
	6–10 years	8	11.9%
	11–20 years	7	10.4%
	Over 20 years	4	6.0%

Notes

*Freq.*Frequency

^a^ This category included the following job titles: Consumer Engagement Coordinator (×2); Manager, Consumer Services (×2); Health Education Officer (×2); Consumer Participation Coordinator; Education Coordinator; Peer Engagement Worker, Peer Support Mentor

### Research question 1. What are the factors that attract individuals to apply for peer worker positions?

Sixty-four participants provided responses to this question. The two most commonly identified themes were: “Using my lived experience to support others” and “To improve the mental health system / make a difference.” Themes identified by three or more participants are listed in Table 2.

**Table 2:** Reasons why participants applied for their current position

Reason	Gov (*N* = 44) n (%)	CMO (*N* = 19) n (%)	Overall (*N* = 64) n (%)
Using my lived experience to support others	23 (52.3%)	10 (52.6%)	34 (53.1%)
To improve mental health system / make a difference	18 (40.9%)	3 (15.8%)	21 (32.8%)
Job fit with my expertise / skill set / qualifications	13 (29.5%)	3 (15.8%)	16 (25.0%)
Wanted to work in mental health / the health system	8 (18.2%)	4 (21.1%)	12 (18.8%)
Challenging / Interesting work^†^	4 (9.1%)	6 (31.6%)	10 (15.6%)
Passion for peer support / mental health	4 (9.1%)	4 (21.1%)	9 (14.1%)
To demonstrate recovery	6 (13.6%)	2 (10.5%)	8 (12.5%)
Working conditions (e.g., working hours, job security, work-life balance)	6 (13.6%)	2 (10.5%)	8 (12.5%)
Build on previous experiences / Improve skills	5 (11.4%)	2 (10.5%)	7 (10.9%)
Did not like previous position	4 (9.1%)	1 (5.3%)	5 (7.8%)
New experiences / something different	2 (4.5%)	3 (15.8%)	5 (7.8%)
Support recovery / recovery oriented practice	4 (9.1%)	1 (5.3%)	5 (7.8%)
To reduce stigma / discrimination	4 (9.1%)	1 (5.3%)	5 (7.8%)
Career progression^†^	0 (0.0%)	3 (15.8%)	3 (4.7%)
Location	2 (4.5%)	1 (5.3%)	3 (4.7%)
To support my own recovery	2 (4.5%)	1 (5.3%)	3 (4.7%)

**Notes:**^†^ Proportions of respondents identifying these elements were significantly different between government and community managed organisation respondents (Pearson’s chi square, *p* < 0.05). Gov = Participants employed in government services; CMO = Participants employed in community managed organisations. Participants could identify more than one reason. Up to eight reasons could be coded

### Research question 2. What are the aspects of work that peer workers like and dislike?

The most common “likes” identified were: “Connecting with consumers”, “Making a difference” and “Positive culture / team relationships”. The most commonly disliked aspects of positions were: “Attitudes of clinicians / Workplace culture”, “Role not valued by others” and “Lack of understanding of the role”. Themes identified by three or more respondents are shown in Tables 3 and 4.

**Table 3:** Aspects of positions liked by respondents

Factor	Gov (*N* = 46) n (%)	CMO (*N* = 20) n (%)	Overall (*N* = 67) n (%)
Connecting with consumers	31 (67.4%)	12 (60.0%)	44 (65.7%)
Making a difference	21 (45.7%)	7 (35.0%)	28 (41.8%)
Positive culture / team relationships	21 (45.7%)	6 (30.0%)	28 (41.8%)
Supporting others’ recovery	15 (32.6%)	7 (35.0%)	22 (32.8%)
Enjoyable, interesting or challenging work	9 (19.6%)	6 (30.0%)	16 (23.9%)
Empowered to deliver meaningful support	8 (17.4%)	2 (10.0%)	10 (14.9%)
Innovation and service improvement	8 (17.4%)	2 (10.0%)	10 (14.9%)
Support from manager / service^†^	10 (21.7%)	0 (0.0%)	10 (14.9%)
Meaningful work	7 (15.2%)	2 (10.0%)	9 (13.4%)
Role valued by others / Pride in role^†^	9 (19.6%)	0 (0.0%)	9 (13.4%)
Supporting my own recovery	4 (8.7%)	3 (15.0%)	7 (10.4%)
Variety	5 (10.9%)	2 (10.0%)	7 (10.4%)
Changing culture	4 (8.7%)	2 (10.0%)	6 (9.0%)
Helping people have a voice and have their needs met	3 (6.5%)	3 (15.0%)	6 (9.0%)
Pay	5 (10.9%)	1 (5.0%)	6 (9.0%)
Flexibility	3 (6.5%)	1 (5.0%)	4 (6.0%)
Self-development and learning	4 (8.7%)	0 (0.0%)	4 (6.0%)
Using my skills and qualifications	2 (4.3%)	2 (10.0%)	4 (6.0%)
Building the peer workforce	3 (6.5%)	0 (0.0%)	3 (4.5%)
Educating others	2 (4.3%)	1 (5.0%)	3 (4.5%)
Mutuality	2 (4.3%)	1 (5.0%)	3 (4.5%)

**Notes:**^†^ Proportions of respondents identifying these elements were significantly different between government and community managed organisation respondents (Pearson’s chi square, *p* < 0.05). Gov = Participants employed in government services; CMO = Participants employed in community managed organisations. Participants could identify more than one reason. Up to eight reasons could be coded

**Table 4:** Aspects of positions disliked by respondents

Factor^†^	Gov (*N* = 46) n (%)	CMO(*N* = 20) n (%)	Overall (*N* = 67) n (%)
Attitudes of clinicians / Workplace culture	16 (34.8%)	6 (30.0%)	22 (32.8%)
Role not valued by others	15 (32.6%)	4 (20.0%)	19 (28.4%)
Lack of understanding of the role	11 (23.9%)	4 (20.0%)	15 (22.4%)
Poor management	7 (15.2%)	6 (30.0%)	13 (19.4%)
Poor pay / Lack of parity in pay / Not enough working hours	7 (15.2%)	5 (25.0%)	12 (17.9%)
Workplace structures preventing optimal practice	6 (13.0%)	4 (20.0%)	10 (14.9%)
High workload / unrealistic expectations	7 (15.2%)	2 (10.0%)	9 (13.4%)
Isolation/ exclusion	7 (15.2%)	1 (5.0%)	8 (11.9%)
Stigma and discrimination / Bullying	4 (8.7%)	4 (20.0%)	8 (11.9%)
Emotional challenges / Supporting consumers experiencing high levels of distress	6 (13.0%)	1 (5.0%)	7 (10.4%)
Lack of role clarity	6 (13.0%)	1 (5.0%)	7 (10.4%)
Poor team dynamics	4 (8.7%)	3 (15.0%)	7 (10.4%)
Distress about the treatment of consumers in the service	6 (13.0%)	0 (0.0%)	6 (9.0%)
Lack of peer workers / small workforce / high turnover	5 (10.9%)	0 (0.0%)	5 (7.5%)
Nothing	4 (8.7%)	1 (5.0%)	5 (7.5%)
Organisational structures - Travel / Location	5 (10.9%)	0 (0.0%)	5 (7.5%)
Lack of career path / opportunities for advancement	3 (6.5%)	1 (5.0%)	4 (6.0%)
Limitations in scope of role	2 (4.3%)	2 (10.0%)	4 (6.0%)
Job instability / insecurity	1 (2.2%)	2 (10.0%)	3 (4.5%)
Organisational structures - meetings / mandatory training	3 (6.5%)	0 (0.0%)	3 (4.5%)
Powerless to raise issues	2 (4.3%)	1 (5.0%)	3 (4.5%)
Reporting lines / lack of appropriate supervision	3 (6.5%)	0 (0.0%)	3 (4.5%)

**Notes:** There were no significant differences proportions of respondents identifying any aspects between government and community managed organisation respondents (i.e., all Pearson’s chi square, *p* > 0.05). Gov = Participants employed in government services; CMO = Participants employed in community managed organisations. Participants could identify more than one aspect. Up to eight aspects could be coded

### Research question 3. What aspects of work lead peer workers to consider leaving their positions?

Thirty-four participants identified factors that had led them to consider leaving their positions. The most common were “Not enough hours / pay” and “Not valued / lack of understanding of the role”. All factors identified are listed in Table 5.

**Table 5:** Reasons for considering leaving position

Reason	Gov (*N* = 23) n (%)	CMO (*N* = 11) n (%)	Overall (*N* = 34) n (%)
Not enough hours / pay	10 (43.5%)	4 (36.4%)	14 (41.2%)
Not valued / lack of understanding of the role	6 (26.1%)	1 (9.1%)	7 (20.6%)
Poor culture / team dynamics	6 (26.1%)	1 (9.1%)	7 (20.6%)
Emotional challenges	5 (21.7%)	1 (9.1%)	6 (17.6%)
Poor management	3 (13.0%)	3 (27.3%)	6 (17.6%)
No opportunity for career advancement	4 (17.4%)	1 (9.1%)	5 (14.7%)
Health reasons	4 (17.4%)	0 (0.0%)	4 (11.8%)
Job insecurity	2 (8.7%)	2 (18.2%)	4 (11.8%)
Organisational barriers to doing my job	2 (8.7%)	2 (18.2%)	4 (11.8%)
Family / work-home balance	2 (8.7%)	1 (9.1%)	3 (8.8%)
Isolation	3 (13.0%)	0 (0.0%)	3 (8.8%)
Lack of support	2 (8.7%)	1 (9.1%)	3 (8.8%)
Not well suited to my strengths	1 (4.3%)	2 (18.2%)	3 (8.8%)
Stigma / discrimination or bullying	2 (8.7%)	1 (9.1%)	3 (8.8%)
To engage in further education / develop other skills	3 (13.0%)	0 (0.0%)	3 (8.8%)
Unable to create change	3 (13.0%)	0 (0.0%)	3 (8.8%)
Want something different	3 (13.0%)	0 (0.0%)	3 (8.8%)
Lack of access to professional development / supervision^†^	0 (0.0%)	2 (18.2%)	2 (5.9%)
Not using my skills	2 (8.7%)	0 (0.0%)	2 (5.9%)
Other	2 (8.7%)	0 (0.0%)	2 (5.9%)

**Notes:**^†^ Proportions of respondents identifying this element were significantly different between government and community managed organisation respondents (Pearson’s chi square, *p* < 0.05). Gov = Participants employed in government services; CMO = Participants employed in community managed organisations. Participants could identify more than one reason. Up to eight reasons could be coded

### Research question 4. How satisfied are peer workers with supervision, professional development and opportunities for career progression?

Fifty-three participants reported receiving supervision. This was delivered by a senior peer worker (*n* = 25), another peer worker at the same level (*n* = 1), a mental health worker (*n* = 21) or “other” (*n* = 6). Mean satisfaction with supervision was 3.75 (standard deviation [SD] = 1.17) on the 5-point scale. After removing the group with only one respondent (receiving supervision from a peer worker at the same level), the ANOVA comparing the remaining three groups revealed a significant between-groups difference (*F*_*(2,48)*_ = 5.45, *p* = 0.007). Post-hoc analyses (Tukey HSD) showed that individuals receiving supervision from “others” were significantly less satisfied with supervision (M = 2.50, SD = 0.84) than those receiving supervision from a senior peer worker (M = 4.13, SD = 0.90). Mean satisfaction for individuals receiving supervision from “a mental health worker” was 3.67 (S.D. = 1.32), which was not significantly different from ratings from the other two groups.

Sixty-six participants reported on satisfaction with professional development and career development opportunities. Mean satisfaction for access to professional development was 3.91 (SD = 1.13) with 50 participants (75.8%) indicating they were satisfied or very satisfied. Mean satisfaction with opportunities for career progression was 2.94 (SD = 1.11) with only 19 participants (28.8%) indicating they were satisfied or very satisfied.

### Research question 5. What levels of job satisfaction, turnover intention and burnout are reported by peer workers and how does this compare with other groups in the mental health workforce?

Descriptive statistics for these variables, as well as results from the independent *t*-tests are shown in Table 6. There were no significant differences between the peer workers and mental health workers from the Scanlan and Still [33] study on any of these measures.

**Table 6:** Job satisfaction, turnover intention and burnout for peer workers and other mental health clinicians

Component	Peer Workers (*n* = 67) Mean (S.D.)	Other mental health clinicians^a^ (*n* = 277) Mean (S.D.)	Independent samples *t*-test results
Job Satisfaction	7.25 (2.08)	6.94 (2.04)	*t* = 1.11, *p* = 0.270
Turnover Intention	1.58 (0.67)	1.46 (0.66)	*t* = 1.31, *p* = 0.192
Disengagement	2.17 (0.50)	2.24 (0.40)	*t* = −1.05, *p* = 0.296
Exhaustion	2.44 (0.50)	2.38 (0.41)	*t* = 1.12, *p* = 0.262

**Notes:***S.D.* Standard Deviation; ^a^Sample drawn from Scanlan and Still [33]

### Research question 6. What are the relationships between various aspects of work experienced by peer workers and job satisfaction, turnover intention and burnout?

Descriptive statistics for the various variables and correlations with job satisfaction, turnover intention, disengagement and exhaustion are shown in Table 7.

**Table 7:** Descriptive statistics and correlation with job satisfaction, turnover intention, disengagement and exhaustion

Component	Potential range	Mean (S.D.)	Correlation with			
			Sat	T.I.	Dis	Exh
Job satisfaction	1 to 10	7.25 (2.08)	–	−0.59^***^	−0.69^***^	−0.42^***^
Turnover Intention	1 to 3	1.58 (0.67)	−0.59^***^	–	0.64^***^	0.56^***^
Disengagement	1 to 4	2.17 (0.50)	− 0.69^***^	0.64^***^	–	0.59^***^
Exhaustion	1 to 4	2.44 (0.50)	−0.42^***^	0.56^***^	0.59^***^	–
**Satisfaction with …**						
*Supervision*	1 to 5	3.56 (1.30)	0.67^***^	−0.52^***^	−0.56^***^	− 0.47^***^
*Professional development*	1 to 5	3.91 (1.13)	0.55^***^	−0.39^**^	−0.53^***^	− 0.28^*^
*Opportunities for career progression*	1 to 5	2.94 (1.11)	0.29^*^	−0.50^***^	−0.29^*^	− 0.32^**^
**Job Demands**						
*Cognitive*	1 to 5	4.28 (0.75)	0.28^*^	−0.05	−0.33^**^	0.17
*Emotional*	1 to 5	4.32 (0.86)	0.01	0.27^*^	−0.02	0.50^***^
*Physical workload*	1 to 5	2.82 (1.24)	−0.29^*^	0.33^**^	0.25^*^	0.39^**^
*Physical environment*	1 to 5	2.41 (1.19)	−0.52^***^	0.43^***^	0.50^***^	0.51^***^
*Recipient contact demands*	1 to 5	3.62 (0.92)	−0.02	0.17	−0.01	0.36^**^
*Shiftwork / Working hours*	1 to 5	2.62 (1.11)	−0.29^*^	0.29^*^	0.22	0.54^***^
*Time pressure*	1 to 5	2.94 (1.05)	0.01	−0.01	−0.13	0.21
*Work-Home interference*	1 to 5	2.23 (1.09)	−0.27^*^	0.28^*^	0.18	0.36^**^
*Workload*	1 to 5	2.71 (1.30)	−0.04	−0.01	− 0.05	0.26^*^
**Job resources**						
*Feedback*	1 to 5	3.67 (1.02)	0.54^**^	−0.39^**^	−0.50^***^	− 0.51^***^
*Job control*	1 to 5	4.04 (0.71)	0.58^***^	−0.29^*^	−0.55^***^	− 0.34^**^
*Job security*	1 to 5	3.45 (1.37)	0.52^***^	−0.38^**^	−0.42^***^	− 0.09
*Participation*	1 to 5	3.35 (1.05)	0.27^*^	−0.12	−0.30^*^	− 0.33^**^
*Rewards and recognition*	1 to 5	3.57 (0.90)	0.63^***^	−0.49^***^	−0.63^***^	− 0.52^***^
*Social support*	1 to 5	4.15 (0.67)	0.46^***^	−0.38^**^	−0.39^**^	− 0.49^***^
*Supervisor support*	1 to 5	3.88 (0.91)	0.50^***^	−0.25	−0.43^**^	− 0.29^*^
*Manager support*	1 to 5	3.68 (1.01)	0.49^***^	−0.48^***^	−0.42^**^	− 0.27^*^

Notes:^*^p < .05, ^**^p < .01, ^***^p < .001; *S.D.* Standard Deviation; *Sat* Job satisfaction; *T.I.* Turnover intention; *Dis* Disengagement; *Exh* Exhaustion

### Research question 7. Are there differences in experiences for those peer workers employed in government and community managed services?

There were no significant differences between peer workers from government and community managed settings in relation to job satisfaction, turnover intention, burnout or satisfaction with supervision, professional development or career progression (Table 8). Additionally, there were very few elements in which there were significant differences in proportions between government and community managed participants in terms of reasons for applying for positions, likes, dislikes and reasons for considering leaving their positions (Tables 2, 3, 4 and 5). Respondents working in community managed organisations were more likely to report that they were attracted to the position due to the challenging / interesting nature of the work and for career progression. Respondents working in government positions were more likely to identify “support from manager / service” and “role valued by others / pride in role” as “likes” of their current position. Lack of access to professional development / supervision was more frequently identified by participants in community managed organisations as being a reason for considering leaving their current position.

**Table 8:** Comparison of ratings between peer workers from government and community managed services

Component	Government services (*n* = 46) Mean (S.D.)	Community managed organisations (*n* = 20) Mean (S.D.)	Independent samples *t*-test results
Job Satisfaction	7.41 (2.07)	6.80 (2.09)	*t* = 1.10, *p* = 0.275
Turnover Intention	4.74 (2.07)	4.85 (1.93)	*t* = − 0.20, *p* = 0.839
Disengagement	2.19 (0.52)	2.18 (0.47)	*t* = 0.04, *p* = 0.971
Exhaustion	2.46 (0.52)	2.41 (0.48)	*t* = 0.36, *p* = 0.722
**Satisfaction with …**			
*Supervision*	3.74 (1.27)	3.06 (1.25)	*t* = 1.86, *p* = 0.068
*Professional development*	3.98 (1.14)	3.75 (1.16)	*t* = 0.74, *p* = 0.462
*Opportunities for career progression*	2.82 (1.03)	3.15 (1.27)	*t* = −1.10, *p* = 0.275

**Notes:***S.D.* Standard Deviation

## Discussion

While this study explored a range of research questions, to our knowledge, it is the first to explore job satisfaction, turnover intention and burnout in a sample of peer workers in mental health services in New South Wales, Australia. When compared with other mental health workers from a previous study undertaken in New South Wales [33] (Research Question 5: “What levels of job satisfaction, turnover intention and burnout are reported by peer workers and how does this compare with other groups in the mental health workforce?”), peer workers’ self-ratings of job satisfaction, turnover intention and burnout were not significantly different. While the relatively small sample size for the current study may have compromised the statistical power of these analyses, exploration of the descriptive statistics (Table 6) also reveals that the differences are of quite small magnitude, especially in relation to disengagement and exhaustion.

Results related to the first research question, “What are the factors that attract individuals to apply for peer worker positions?” are perhaps unsurprising. The most commonly-reported factors were “using my lived experience to support others” and “to improve the mental health system / make a difference”. Using lived experience to support others is a unique characteristic of peer worker positions when compared with other roles in mental health services. The altruistic desire to support others facing similar challenges is common amongst many individuals who have faced and overcome serious health concerns and/or trauma [45] and peer work roles provide formal opportunities for this to happen, as well as the opportunity “to demonstrate recovery” (i.e., to be a role model and be an example that recovery is possible). Additionally, one of the key benefits of increasing the numbers of peer workers in mental health services has been identified as promoting a stronger recovery orientation, which has been suggested to have a potentially transformative effect on mental health service delivery [1, 4, 46]. Participants’ responses in this study highlight that this opportunity to improve the mental health system (and to “support recovery / recovery oriented practice”) is an important attraction to working in peer worker roles.

Similarly, results from the first part of Research Question 2 (“What are the aspects of work that peer workers like?”) highlight the importance of interpersonal connection, “making a difference” and supporting others’ recovery. Other “positive” aspects of work roles related to peer workers’ relationships with other members of mental health teams. “Positive culture / team relationships” was identified as a positive aspect of work by over 40% of respondents. While reported by a lower proportion of respondents, factors such as “support from manager / service”, “role valued by others” and “changing culture” were all identified as positive aspects of positions. Results from the second part of Research Question 2 (“What are the aspects of work that peer workers dislike?”) also highlight the importance of team relationships, albeit from a more negative perspective. The four most commonly-reported “dislikes” about positions were related to relationships amongst the team, with the most common being “attitudes of clinicians / workplace culture” and “role not valued by others.” Other negative aspects of positions included lack of connections with other peer workers, a sense of isolation / exclusion and high workloads, often related to unrealistic expectations from others, expectations to fill gaps or complete menial tasks.

While negative aspects of positions can cause dissatisfaction, these can also drive individuals to consider leaving their positions. Responses related to Research Question 3 (“What aspects of work lead peer workers to consider leaving their positions?”) further highlight those factors. While challenges with other members of the team / management were prominent in responses, the issues of “not enough hours / pay”, “emotional challenges” and “no opportunity for career advancement” were also identified.

Considering participants’ responses related to aspects of work that were disliked and reasons for considering quitting, these are very similar to the challenges identified by peer workers in previous studies as described in the introduction. Poor attitudes / team culture; lack of respect for the role, the person or their abilities; lack of role clarity; insufficient working hours or pay; and emotional challenges have all been highlighted in previous literature [10, 11, 13, 15, 16, 19]. However, the issue of lack of opportunities for career advancement has been identified less frequently [22].

Research Question 4 (“How satisfied are peer workers with supervision, professional development and opportunities for career progression?”) enabled further exploration of the issue of opportunities for career progression. Mean satisfaction with “opportunities for career progression” was 2.94 on a 5-point scale. Being below the mid-point of the scale, this suggests that there is overall dissatisfaction with opportunities for career progression. The proportion of individuals who were “satisfied” or “very satisfied” with opportunities for career progression in this study (28.8%) was lower than in a study of occupational therapists in mental health [34] where 43.8% of respondents reported that they felt that there were good opportunities for career progression.

Other results related to Research Question 4 suggest that while most participants were satisfied with opportunities for professional development and access to supervision, those individuals who did not receive supervision from a senior peer worker were less satisfied with supervision than those who did. This difference was statistically significant for individuals receiving supervision from “others” which included a chaplain, a project officer and managers / team leaders who did not identify as having lived experience of mental health issues. Satisfaction with supervision had moderate correlations (*r* > 0.5) with overall job satisfaction, lower turnover intention and lower levels of disengagement. Satisfaction with professional development had moderate correlations with overall job satisfaction and lower levels of disengagement. Lower satisfaction with opportunities for career progression had a moderate correlation with higher levels of turnover intention. These results suggest that strengthening access to quality supervision from a senior peer worker, professional development and opportunities for career progression may support the workplace experiences of consumer peer workers.

Results related to lack of opportunities for career progression, lack of connection with other peer workers and difficulties accessing support and supervision highlight the importance of lived experience leadership and of managers having a strong understanding of the role and importance of peer work [10, 20, 21, 24, 25]. While further training for leaders, managers and non-peer mental health workers is essential [21, 24], expansion of the peer workforce, especially an increase in the numbers of senior peer workers, could go some way towards addressing these issues. An increase in the number of senior peer workers would enable more peer workers to access supervision from a senior and would also provide opportunities for career advancement. Increasing the overall numbers of peer workers may also address concerns about high workloads, inability to access sufficient work hours and would provide greater opportunities for connections between peer workers to reduce the sense of isolation felt by some. An overall increase in the number of peer workers in the mental health system may also, over time, help to shift clinician attitudes and support a sense that the role of peer workers is valued [25].

Results related to Research Question 6 (“What are the relationships between various aspects of work experienced by peer workers and job satisfaction, turnover intention and burnout?”) enable the further exploration of which factors are the most important correlates of job satisfaction, turnover intention and burnout for this group of peer workers. Several of the key findings are discussed below.

Consistent with the theory underpinning the Job-Demands Resources model [41, 42], job emands were most strongly associated with the exhaustion element of burnout for participants in this study. However, the job demand of physical environment was also associated with lower job satisfaction, higher turnover intention and higher disengagement. Notably, the job demands of cognitive demands and time pressure did not demonstrate the typical patterns of associations. Time pressure did not show correlations with any of the main outcomes and cognitive demands actually showed a positive correlation with job satisfaction and a negative correlation with disengagement (suggesting higher levels of cognitive demands were weakly associated with more positive workplace experiences). This is consistent with Ven den Broeck et al.’s [47] extension of the Job-Demands Resources model in which Job Demands are separated into job challenges and job hindrances. This extension proposes that job challenges (such as cognitive demands and time pressure) can actually be engaging when they are not present at extreme levels [47].

Job Resources tended to be more consistently associated with all outcomes, although tended to be more strongly associated with Job Satisfaction and lower levels of disengagement. Social support, job control, feedback, rewards and recognition, supervisor support and manager support were all associated with more positive overall workplace experiences.

Similar issues surrounding lack of autonomy, lack of social support and issues with the physical environment (e.g., not having places to meet with consumers, not having a desk or computer, noisy or otherwise unpleasant work spaces and, potentially, working in environments which are restrictive in nature) have been raised in previous studies [10, 12, 15, 16, 19]. Interestingly, these results are somewhat different to those reported in the previous study of other groups within the mental health workforce [33], suggesting that consumer peer workers may face different challenges in terms of accessing support, having autonomy in their work and having access to work spaces. Being denied access to basic resources such as a desk and computer and lack of autonomy (job control) may be representative of a lack of valuing of the contribution and professionalism of consumer peer workers [10, 13, 16–18] and lack of social support could represent a sense of isolation [12, 13, 15, 19] . This may highlight an area for consideration when peer workers are employed as the sole peer worker in a team or service and suggests further opportunities for networking and building communities of practice among the peer workforce could be beneficial to improve social support. Peer networking and communities of practice may also strengthen satisfaction with professional development which may have additional benefits in terms of satisfaction and engagement.

The assertion that people with lived experience of mental health issues and recovery are “too emotionally fragile” to cope with the challenges of work in mental health has previously been held up as a “myth” [4]. Overall results from this study give further evidence that this assertion is indeed a “myth”. Participants’ self-ratings of burnout in this study were no higher than those reported in previous studies of other groups of mental health workers. Additionally, while emotional demands were rated highly, these were not rated substantially higher by peer workers in comparison to other mental health workers. Peer workers also identify numerous emotionally challenging issues that are not typically raised by other mental health workers (e.g., negative attitudes of clinicians / workplace cultures; stigma and discrimination; and distress about the treatment of consumers in the service). Given this, the fact that they do not report lower levels of job satisfaction and higher levels of turnover intention and burnout when compared with other mental health workers might suggest that peer workers may require and apply levels of resilience beyond that which is required by other mental health workers. However, given the nature of the results, this interpretation can only be speculative, as there may be numerous other explanations.

Two additional issues prominent in the findings from this study relate to challenges associated with perceptions of other mental health workers in a lack of valuing the role of peer workers. While it is not uncommon for other professional groups in mental health to feel that their roles are not valued (e.g., [48–50]), many of the qualitative comments received in this study suggest that this “lack of valuing” of the role of peer workers may be more prevalent and sometimes more overt than it is for other disciplines. Comments suggested that some non-peer mental health workers believe that peer workers are incapable of providing valuable support to consumers given peer workers’ experience of mental illness or that there is no value in the kinds of support that can be offered by peer workers. This, combined with negative attitudes of clinicians (both towards peer workers and towards consumers more generally), highlights that further work is needed – both in terms of promoting better understanding of the role of peer work and promoting more recovery-oriented attitudes in the mental health workforce. While peer workers are important agents for this change, this change also needs to be supported from other areas within mental health services. If peer workers are the lone voices for change, often having to advocate against exclusionary policies, processes, organisational culture and beliefs, this can lead to perceptions of peer workers as creating disharmony and turbulence and being labelled as disruptive or “problem-makers”. Additionally, peer workers often choose to avoid voicing their concerns for fear of retribution or embarrassment.

### Limitations

There are a number of limitations that should be taken into consideration when interpreting the results from this study. First of these is the fact that the sample is not random, therefore there may be a sample bias. Perhaps those peer workers with the poorest workplace experiences selected not to participate, or alternatively, those peer workers who were most engaged and enjoying their work felt there was no reason to participate in the study. Secondly, the sample size was relatively small and it was not possible to determine a true response rate as reliable information about the number of consumer peer workers working in New South Wales was not available. This means that for some analyses, the study may not be sufficiently powered to detect differences. Thirdly, as the data were cross-sectional in nature, no causal relationships can be inferred from associations found in this study. Additionally, as the study was undertaken in only one state of Australia, the results from this study may not generalise to other contexts in other states or countries. While the comparison between consumer peer workers and other mental health staff ratings in terms of job satisfaction, turnover intention and burnout is useful, it should be recognised that these samples have a number of important differences, most notably that the sample of other mental health workers was drawn from one metropolitan, government mental health service. This means that the two samples may not be directly comparable. Finally, while there were advantages identified in asking open-ended questions about attractions, likes, dislikes and reasons for considering quitting, there are also disadvantages. If all participants responded to the same pre-determined lists of factors / issues, then it would have made direct comparison between participants easier. In the current design, it is not possible to determine if each respondent listed all relevant factors or only those that were most salient or memorable at the time of completing the survey.

## Conclusion

This study is, to our knowledge, the first to explore job satisfaction, turnover intention, burnout and job demands and resources in a sample of peer workers. Results suggest that peer workers are not less satisfied nor more burnt out than mental health professionals from other disciplines. However, results do suggest that more needs to be done to support the full integration and acceptance of the peer workforce. Key areas for improvement include the expansion of the peer workforce (especially senior peer workers who can provide supervision, leadership and systemic advocacy) and continuing education of the non-peer mental health workforce to understand the unique skills and contribution of peer workers and to promote more inclusive attitudes.

Future research could build on these results by extending the research across Australia. To support such a study, a copy of the survey instrument (modified to be relevant for across Australia and adding a list of responses for questions related to attractions, likes and dislikes) has been included as a Supplemental file.

## Supplementary information

**Additional file 1.**

## Data Availability

The datasets used and/or analysed during the current study are available from the corresponding author on reasonable request.

## Abbreviations

CMO: Community Managed Organisation
FTE: Full-time equivalent
MBI: Maslach Burnout Inventory
OBLI: Oldenburg Burnout Inventory
