# Supplementary material for: Workplace experiences of mental health consumer peer workers in New South Wales, Australia: a survey study exploring job satisfaction, burnout and turnover intention
Source: BMC Psychiatry. 2020 Jun 1;20:270. doi: 10.1186/s12888-020-02688-9 (PMC7268613; doi:10.1186/s12888-020-02688-9)
Supplement: Supplementary file 1 — Additional file 1. [file 12888_2020_2688_MOESM1_ESM.pdf]

Workforce survey of consumer workers / peer workers in mental health services

This survey is designed to explore the workplace experiences of individuals with lived experience of mental illness and recovery who work in designated peer worker / consumer worker roles. This will help to understand the enjoyable and challenging aspects of these roles and to make recommendations that might improve the workplace experiences of peer workers / consumer workers.

While we recognise that many people who work in the mental health system have their own experience of mental illness and recovery (and often use this as an aspect of their role), this study is particularly interested in exploring the experiences and perspectives of individuals who are working in identified peer / consumer roles.

This questionnaire will take approximately 30 minutes to complete.

Your participation in this project is entirely voluntary. Your choice to participate or not will have no impact on your relationships with others in the organisation.

INCLUSION CRITERIA

Are you currently employed in an identified peer worker / consumer worker position. That is, a position where "lived experience of mental illness and recovery" (or another similar criterion) is a specific selection criterion?	☐ Yes	☐ No
Do you work in Australia?	☐ Yes	☐ No

If you answered "**Yes**" to both questions, please proceed. If you answered **"No"** to either question, thanks for your interest, but you are not eligible for participation in this study.

INFORMATION ABOUT YOU

What is your gender / gender identity?

What is your age?

☐ 25 or under ☐ 26-30 ☐ 31-40 ☐ 41-50
☐ 51-60 ☐ 61-70 ☐ 71 or over ☐ I'd rather not say

Which state / territory do you work in

☐ Australian Capital Territory ☐ New South Wales
☐ Northern Territory ☐ Queensland
☐ South Australia ☐ Tasmania
☐ Victoria ☐ Western Australia

Is the location where you work:

☐ Metropolitan ☐ Regional
☐ Rural ☐ Remote

Developed by Justin Scanlan, Megan Still, Jae Radican, Daya Henkel, Tim Heffernan, Peter Farrugia, Jemima Isbester and Jessica English. (contact justin.scanlan@sydney.edu.au)

INFORMATION ABOUT YOUR CURRENT PEER / CONSUMER WORKER POSITION

(If you happen to have more than one role, please respond in terms of thinking about your "primary" or "main" role. If your experiences in different roles are very different, we would love it if you would be so kind as to complete the survey for each of your roles).

Is your position in a government, non-government / community managed organisation or in the private sector?

- ☐ Government Health Services (e.g., NSW Health, Qld Health, SA Health etc)
- ☐ Non-government / community managed organisation
- ☐ Private sector
- ☐ Other (please describe): ___________________

What is your current position title (as stated on your employment contract)?

Does this position title reflect your job role accurately? ☐ Yes ☐ No

If no, please describe why:

Over the last 3 months, how many hours per week (on average) were you paid for? _______ ***hours***

Over the last 3 months, how many hours per week (on average) did you actually work? _______ ***hours***

In terms of your current working hours, would you say:

- ☐ I would prefer to work more hours
- ☐ My working hours are about right
- ☐ I would prefer to work fewer hours

If you answered that you would prefer to work more hours or fewer hours, please briefly explain the reasons below

Is your current role

- ☐ Permanent
- ☐ Temporary / Fixed-term contract
- ☐ Casual
- ☐ I don't know

Is your current position focused on inpatient or community services?

- ☐ Totally / almost totally community-based
- ☐ Mainly community but some inpatient work
- ☐ About an even split between community and inpatient
- ☐ Mainly inpatient but some community work
- ☐ Totally / almost totally inpatient-based

Please describe the mix of workers in the team you work in

- ☐ I work with only other peer / consumer workers
- ☐ I work with mainly other peer / consumer workers and a few clinicians / mental health workers
- ☐ I work about an even mix of peer / consumer workers and clinicians / mental health workers
- ☐ I work with mainly clinicians / mental health workers and a few other peer / consumer workers
- ☐ I work with only clinicians / mental health workers

Comments:

How long have you been working in your <u>current</u> peer / consumer worker role?

☐ Less than 1 year	☐ 1-2 years	☐ 3-5 years
☐ 6-10 years	☐ 11-20 years	☐ Over 20 years

In total, how long have you worked in peer / consumer worker roles?

☐ Less than 1 year	☐ 1-2 years	☐ 3-5 years
☐ 6-10 years	☐ 11-20 years	☐ Over 20 years

Have you worked in other (non peer / consumer worker) roles in mental health?

- ☐ Yes
- ☐ No

If yes, what was that role / were those roles (if you've held several other positions)?

In regard to your previous role/s, would you say that they were:

- ☐ Quite similar to my role as a peer / consumer worker
- ☐ Sort of similar, sort of different
- ☐ Quite different to my role as a peer / consumer worker

Any comments about similarities or differences?

Were you employed prior to coming in to your current position?

☐ **Yes** ☐ **No** →

If no, how long had you been out of work prior to this position?

If yes, what was your previous position?

- ☐ As described in the previous question
- ☐ Other (please describe):

Why did you decide to leave that position?

Please continue onto the next page

QUESTIONS ABOUT YOUR CURRENT POSITION

Overall, on a scale of 1 to 10, how satisfied are you with your current job? (Circle the most appropriate number)

circle your response ⇨	1	2	3	4	5	6	7	8	9	10
	Very dissatisfied				*Neither satisfied nor dissatisfied*					*Very satisfied*

Please indicate how important each one of these factors were in you deciding to apply for your current position

	Not applicable	Not at all important	Somewhat important	Quite important	Extremely important
Challenging / Interesting work	☐	☐	☐	☐	☐
Did not like previous position	☐	☐	☐	☐	☐
For career progression	☐	☐	☐	☐	☐
I needed / wanted a job	☐	☐	☐	☐	☐
Job fit with my expertise / skill set / qualifications	☐	☐	☐	☐	☐
Location	☐	☐	☐	☐	☐
New experiences / something different	☐	☐	☐	☐	☐
Passion for peer support / mental health	☐	☐	☐	☐	☐
Physical health focus	☐	☐	☐	☐	☐
Support recovery / recovery oriented practice	☐	☐	☐	☐	☐
Team work	☐	☐	☐	☐	☐
To build on previous experiences / Improve skills	☐	☐	☐	☐	☐
To demonstrate recovery	☐	☐	☐	☐	☐
To improve mental health system / make a difference	☐	☐	☐	☐	☐
To reduce stigma / discrimination	☐	☐	☐	☐	☐
To support my own recovery	☐	☐	☐	☐	☐
Using my lived experience to support others	☐	☐	☐	☐	☐
Wanted to work in mental health / the health system	☐	☐	☐	☐	☐
Working conditions (e.g., working hours, job security, work-life balance)	☐	☐	☐	☐	☐
Other (please describe): ____________________	☐	☐	☐	☐	☐
Other (please describe): ____________________	☐	☐	☐	☐	☐
Other (please describe): ____________________	☐	☐	☐	☐	☐
Other (please describe): ____________________	☐	☐	☐	☐	☐

Please indicate how positive each of the following aspects are in relation to your current job

	Not at all positive	A little bit positive	Moderately positive	Quite positive	Very positive
Autonomy	☐	☐	☐	☐	☐
Building the peer workforce	☐	☐	☐	☐	☐
Changing culture	☐	☐	☐	☐	☐
Connecting with consumers	☐	☐	☐	☐	☐
Educating others	☐	☐	☐	☐	☐
Empowered to deliver meaningful support	☐	☐	☐	☐	☐
Enjoyable, interesting or challenging work	☐	☐	☐	☐	☐
Flexibility	☐	☐	☐	☐	☐
Helping people have a voice and have their needs met	☐	☐	☐	☐	☐
Innovation and service improvement	☐	☐	☐	☐	☐
Making a difference	☐	☐	☐	☐	☐
Meaningful work	☐	☐	☐	☐	☐
Mutuality	☐	☐	☐	☐	☐
Pay	☐	☐	☐	☐	☐
Positive culture / team relationships	☐	☐	☐	☐	☐
Role valued by others / Pride in role	☐	☐	☐	☐	☐
Self-development and learning	☐	☐	☐	☐	☐
Support from manager / service	☐	☐	☐	☐	☐
Supporting my own recovery	☐	☐	☐	☐	☐
Supporting others' recovery	☐	☐	☐	☐	☐
Using my skills and qualifications	☐	☐	☐	☐	☐
Variety	☐	☐	☐	☐	☐
Other (please describe): ______________________	☐	☐	☐	☐	☐
Other (please describe): ______________________	☐	☐	☐	☐	☐
Other (please describe): ______________________	☐	☐	☐	☐	☐
Other (please describe): ______________________	☐	☐	☐	☐	☐

Please indicate how negative each of the following aspects are in relation to your current job

	Not at all negative	A little bit negative	Moderately negative	Quite negative	Very negative
Attitudes of clinicians / Workplace culture	☐	☐	☐	☐	☐
Distress about the treatment of consumers in the service	☐	☐	☐	☐	☐
Emotional challenges / Supporting consumers experiencing high levels of distress	☐	☐	☐	☐	☐
High workload / unrealistic expectations	☐	☐	☐	☐	☐
Isolation/ exclusion	☐	☐	☐	☐	☐
Job instability / insecurity	☐	☐	☐	☐	☐
Lack of career path / opportunities for advancement	☐	☐	☐	☐	☐
Lack of engagement by consumers	☐	☐	☐	☐	☐
Lack of peer workers / small workforce / high turnover	☐	☐	☐	☐	☐
Lack of role clarity	☐	☐	☐	☐	☐
Lack of understanding of the role	☐	☐	☐	☐	☐
Limitations in scope of role	☐	☐	☐	☐	☐
Organisational structures - meetings / mandatory training	☐	☐	☐	☐	☐
Organisational structures - Travel / Location	☐	☐	☐	☐	☐
Physical work environment	☐	☐	☐	☐	☐
Poor management	☐	☐	☐	☐	☐
Poor pay / Lack of parity in pay / Not enough working hours	☐	☐	☐	☐	☐
Poor team dynamics	☐	☐	☐	☐	☐
Powerless to raise issues	☐	☐	☐	☐	☐
Qualifications not valued	☐	☐	☐	☐	☐
Reporting lines / lack of appropriate supervision	☐	☐	☐	☐	☐
Role not valued by others	☐	☐	☐	☐	☐
Stigma and discrimination / Bullying	☐	☐	☐	☐	☐
Workplace structures preventing optimal practice	☐	☐	☐	☐	☐
Other (please describe): ______________________	☐	☐	☐	☐	☐
Other (please describe): ______________________	☐	☐	☐	☐	☐
Other (please describe): ______________________	☐	☐	☐	☐	☐
Other (please describe): ______________________	☐	☐	☐	☐	☐

The next questions are related to whether you are currently considering leaving your current position.

	Yes	*Unsure*	*No*
I am actively looking for another job	☐	☐	☐
As soon as I can find another job, I will quit	☐	☐	☐
I often think about quitting my job	☐	☐	☐
If you are considering quitting, what are the main reasons for this?			

INFORMATION ABOUT YOUR SUPPORT AND TRAINING

Have you completed peer-specific training or other mental health worker training (tick all that apply)?

- ☐ Certificate IV in Mental Health Peer Work
- ☐ Certificate IV in Mental Health
- ☐ Intentional Peer Support
- ☐ Purposeful storytelling
- ☐ A health professional qualification (please specify if you are comfortable doing so):

____

- ☐ Other (please specify below)

____

____

____

Who do you currently receive Professional Development Supervision from?

- ☐ A Senior Peer Worker / Consumer worker
- ☐ Another peer worker / consumer worker at the same level as I am
- ☐ A mental health worker who is not a peer worker / consumer worker
- ☐ Other (please specify below):

____

- ☐ I do not receive Professional Development Supervision

How satisfied are you with your current Professional Development Supervision arrangements

- ☐ Very satisfied
- ☐ Satisfied
- ☐ Neither satisfied nor dissatisfied
- ☐ Dissatisfied
- ☐ Very dissatisfied
- ☐ Not applicable (I don't receive Professional Development Supervision)

Please comment on this rating:

How satisfied are you with your current access to training and other professional development opportunities (not including Professional Development Supervision)?

- ☐ Very satisfied
- ☐ Satisfied
- ☐ Neither satisfied nor dissatisfied
- ☐ Dissatisfied
- ☐ Very dissatisfied

Please comment on this rating:

How satisfied are you with your current opportunities for career progression?

- ☐ Very satisfied
- ☐ Satisfied
- ☐ Neither satisfied nor dissatisfied
- ☐ Dissatisfied
- ☐ Very dissatisfied

Please comment on this rating:

The next questions are related to your perception of a variety of characteristics of your current position

The first set of questions relate to **your manager**. In this survey, a “manager” is the person who monitors your work performance, approves leave and authorises your pay. If you do not have a manager, please select “I do not have a manager” and move on to the next question.

☐ I do not have a manager. Please move on to the next question. (Please tick the box and then move on to the next question)

	Strongly disagree	*Disagree*	*Neutral*	*Agree*	*Strongly Agree*
My achievements are recognised by my manager	☐	☐	☐	☐	☐
My manager is concerned about the welfare of the people that work for them.	☐	☐	☐	☐	☐
My manager inspires me to do my best work	☐	☐	☐	☐	☐
My manager uses their influence to help me solve problems	☐	☐	☐	☐	☐

The next set of questions relate to **your Professional Development Supervisor**. In this survey, “Professional Development Supervisor” is the person who supports you to reflect on your work and assist you to apply your lived experience in a purposeful way in your role. If you do not have a Professional Development Supervisor, please select “I do not have a Professional Development Supervisor” and move on to the next question. If your supervisor is the same person as your manager, please select “My Professional Development Supervisor is the same person as my manager” and then move on to the next question.

☐ I do not have a Professional Development Supervisor. (Please tick the box and then move on to the next question.)

☐ My Professional Development Supervisor is the same person as my manager. (Please tick the box and then move on to the next question.)

	Strongly disagree	*Disagree*	*Neutral*	*Agree*	*Strongly Agree*
My achievements are recognised by my supervisor	☐	☐	☐	☐	☐
My supervisor is concerned about the welfare of the people that work for them	☐	☐	☐	☐	☐
My supervisor inspires me to do my best work	☐	☐	☐	☐	☐
My supervisor uses their influence to help me solve problems	☐	☐	☐	☐	☐

Please rate your level of agreement with each of the following statements in relation to your current position:

	Strongly disagree	*Disagree*	*Neutral*	*Agree*	*Strongly Agree*
The job requires me to keep track of more than one thing at a time	☐	☐	☐	☐	☐
My work requires a lot of concentration	☐	☐	☐	☐	☐
The job gives me a chance to use my personal initiative or judgment in carrying out the work	☐	☐	☐	☐	☐
Only the management decides what everybody has to do	☐	☐	☐	☐	☐
My work is emotionally demanding	☐	☐	☐	☐	☐
The job gives me considerable opportunity for independence and freedom in how I do the work	☐	☐	☐	☐	☐
I always have enough time to perform my tasks	☐	☐	☐	☐	☐
I receive feedback on my performance from other people in my organization (such as my manager or coworkers)	☐	☐	☐	☐	☐
I get enough feedback about the quality of my performance	☐	☐	☐	☐	☐
My colleagues are willing to give me help if I ask for it	☐	☐	☐	☐	☐
My job is physically taxing	☐	☐	☐	☐	☐
It is physically taxing for me to get used to my working times	☐	☐	☐	☐	☐
My achievements are recognised by my colleagues	☐	☐	☐	☐	☐
My performance is rewarded properly	☐	☐	☐	☐	☐
The job allows me to make my own decisions about how to schedule my work	☐	☐	☐	☐	☐
My working hours allow me to meet my family and personal obligations	☐	☐	☐	☐	☐
My physical working conditions – climate, light, noise, design of the working place, and material – are alright	☐	☐	☐	☐	☐
My job requires working very hard or very fast	☐	☐	☐	☐	☐
I feel my job is secure	☐	☐	☐	☐	☐
My contact with persons to whom I offer services is demanding	☐	☐	☐	☐	☐
I have good relationships with my colleagues	☐	☐	☐	☐	☐

If you would like to explain any of your responses to the items above, please do so in the box below:

The final set of questions is related to your feelings about your current work.

	Strongly disagree	*Disagree*	*Agree*	*Strongly agree*
I always find new and interesting aspects in my work.	☐	☐	☐	☐
There are days when I feel tired before I arrive at work.	☐	☐	☐	☐
It happens more and more often that I talk about my work in a negative way.	☐	☐	☐	☐
After work, I tend to need more time than in the past in order to relax and feel better.	☐	☐	☐	☐
I can tolerate the pressure of my work very well.	☐	☐	☐	☐
Lately, I tend to think less at work and do my job almost mechanically.	☐	☐	☐	☐
I find my work to be a positive challenge.	☐	☐	☐	☐
During my work, I often feel emotionally drained.	☐	☐	☐	☐
Over time, one can become disconnected from this type of work.	☐	☐	☐	☐
After working, I have enough energy for my leisure activities.	☐	☐	☐	☐
Sometimes I feel sickened by my work tasks.	☐	☐	☐	☐
After my work, I usually feel worn out and weary.	☐	☐	☐	☐
This is the only type of work that I can imagine myself doing.	☐	☐	☐	☐
Usually, I can manage the amount of my work well.	☐	☐	☐	☐
I feel more and more engaged in my work.	☐	☐	☐	☐
When I work, I usually feel energized.	☐	☐	☐	☐

If you would like to explain any of your responses to the items above, please do so in the box below:

If you have any final comments about your overall experiences in your peer worker / consumers worker position, please list these in the box below.

Thank you for completing this questionnaire.

If completing this questionnaire has raised any issues which are concerning or that you would like to discuss, you can access free, professional and strictly confidential counselling and support services through the Employee Assistance Program at your workplace or Lifeline on 13 11 14.
